# The role of family confidants and caregivers in the care of older cancer patients: Extending the concept of “shared decision‐making”

**DOI:** 10.1002/hsr2.281

**Published:** 2021-05-06

**Authors:** Frank Gieseler, Andreas Heidenreich, Jacqueline Schubert, Fabian Frielitz, Christoph Rehmann‐Sutter, Frank Wörler, Christina Schües, Joachim Hübner, Susanne Elsner, Katarina Block, Achim Rody, Nikolas von Bubnoff, Tobias Keck, Monika Steimann, Gero Endsin, Alexander Katalinic

**Affiliations:** ^1^ Clinic for Hematology and Oncology University Hospital Schleswig‐Holstein (UKSH) Luebeck Germany; ^2^ Institute for Social Medicine and Epidemiology, University of Luebeck Luebeck Germany; ^3^ Institute for History of Medicine and Science Studies, University of Luebeck Luebeck Germany; ^4^ Clinic for Gynecology University Hospital Schleswig‐Holstein (UKSH) Luebeck Germany; ^5^ Clinic for Surgery University Hospital Schleswig‐Holstein (UKSH) Luebeck Germany; ^6^ Strandklinik Ostseebad Boltenhagen Ostseebad Boltenhagen Germany; ^7^ VAMED, Rehaklinik Lehmrade Lehmrade Germany

**Keywords:** family, older cancer patients, qualitative research methods, quality of life, survivorship

## Abstract

**Background and aims:**

Family caregivers play an important role in assisting their family members with cancer, but their influence on the treatment decision‐making process has not yet been adequately investigated. This exploratory study approached this topic via reconstructive methodology, focusing on assessing patient‐caregiver relationships.

**Methods:**

We conducted semi‐structured interviews with 37 mostly elderly cancer patients (median age: 74 years) about the context of their diagnosis, treatment decision, and family support. Additionally, we interviewed 34 caregivers of cancer patients. Of these, 25 were related to patients interviewed. We analyzed the interviews via a multi‐step coding method informed by Grounded Theory methodology toward characterizing patient‐caregiver relationships, the treatment decision‐making process, and the caregivers' role therein.

**Results:**

In the majority of cases (86%), patients were being supported by caregivers. We categorized patient‐caregiver relationships in regards to the caregivers' involvement in the therapy decision‐making process. We found patient‐caregiver interaction patterns that indicate the potential of caregivers to decidedly influence the therapy decision‐making process. Yet, only in 38% of cases, a caregiver attended relevant patient‐physician‐consultations.

**Conclusion:**

Depending on the nature of the patient‐caregiver relationship, the traditional concept of shared decision‐making, which assumes a dyadic relationship, needs to be extended toward a more dynamic concept in which caregivers should be involved more frequently. This could enable physicians to better understand a patient's reasons for or against a therapy proposal and ensure that the patient's wishes are communicated and considered. On the other hand, strong caregiver‐involvement bears risks of over‐stepping elderly patients' wishes, thus violating patient autonomy.

## 1 INTRODUCTION AND BACKGROUND

The care of cancer patients of advanced age requires special caution due to age‐associated limitations of organ functions, co‐morbidities, and co‐medication.^1^, ^2^ New pharmacological developments, however, represent increased therapeutic options also for older patients.^3^, ^4^ These developments, combined with the demographic shift to more elderly patients, underline the need for sensitive and reasonable patient/doctor conversations that include a clear understanding of the treatment goals by both parties involved.

Several studies have shown that the adherence of patients to their doctors' treatment recommendations is surprisingly low. This is especially true of elderly cancer patients and oral cancer therapy, with adherence rates down to 46%.^5^ Good physician and patient communication was identified as one of the major contributing factors for adherence.^6^, ^7^ One of the reasons for the complexity of patient/doctor talks especially with older patients is the multilayer situation of rational decision‐making regarding the proposed therapy in a frightening situation in which a patient is facing a potentially deadly illness.^8^, ^9^

Family confidants and caregivers (CGs) play an important role in supporting their ill family members in many respects. Some of their major duties are managing the home and housekeeping and support in domestic chores including preparing and assisting with meals, dealing with mobility issues, arranging for transportation to medical care facilities, making medical appointments, managing prescription medication and also offering emotional support. However, the extent of CGs' influence on patients' decision‐making regarding therapy recommendations given by the oncologist is not clear. Therefore, we conducted a study in which we analyzed interviews with older cancer patients (median: 74 years) and their CGs, focusing on the patterns of relationships between them.

## 2 METHODS

**FIGURE 1:**
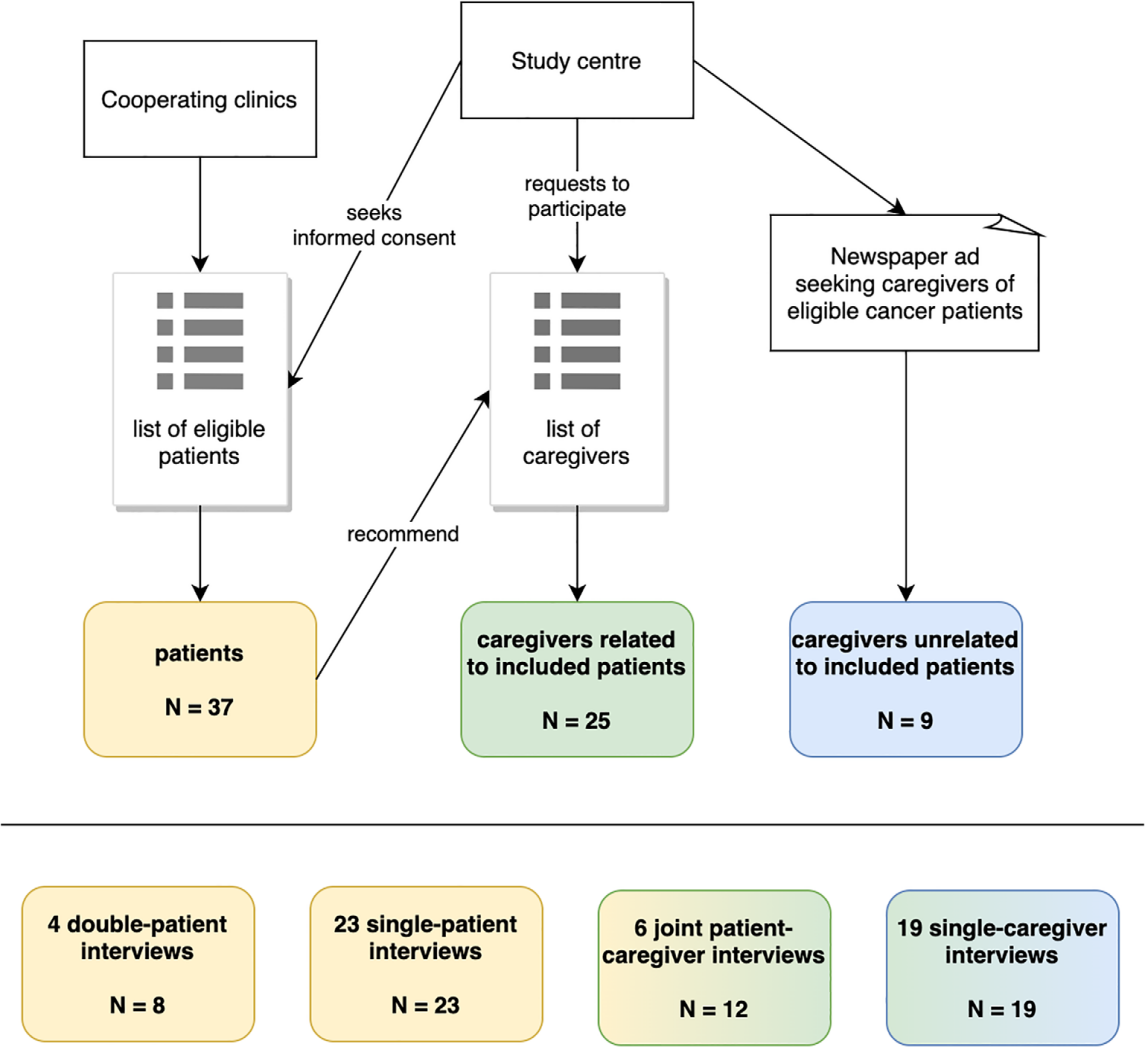
Illustration of the interview recruitment process and interview settings

We conducted semi‐structured,^10^, ^11^ guideline‐based interviews^12^ with 37 mostly elderly (55‐89 years, median age: 74 years) recently diagnosed cancer patients and 34 CGs, most of whom were related to the patients interviewed. Cancer patients were recruited in a German cancer center and two rehabilitation clinics and asked for contacts to persons who had supported them during the course of their illness. As additional patients' recruitment would have posed a burden for the clinics, we opted for an alternative recruitment path to address the CGs of elderly cancer patients directly. Interview guidelines were developed in an interdisciplinary process with the collaboration of oncologists, social scientists, and philosophers based on the SPSS‐method, according to Helfferich.^12^, ^13^ After a pre‐test with four patients and two CGs, the interview guidelines were deemed suitable and understandable. Interviews were held in various settings between November 2017 and May 2018 (see Figure 1 for the participant‐recruitment process and interview settings, Table 1 for the relevant patient and CG characteristics).

**TABLE 1:** Patient and caregiver characteristics

Patient characteristics (n = 37)	
Age	
Median [range]	74 [55‐89]
Sex	
Male	6
Female	31
Caregiver characteristics (n = 34)	
Sex	
Male	14
Female	20
Relationship to patient	
Husband	9
Son	2
Son‐in‐law	–
Brother	1
Wife	5
Daughter	12
Daughter‐in‐law	1
Sister	2
Friend	2

The interviewers prompted the participants to give free and in‐depth answers, as our approach was exploratory. The notion that CGs are an important group in the context of elderly cancer patients' illness did not only emerge during the analysis of patient interviews. As we did not pursue a preconceived research question and did not a priori focus on a particular dimension, we did not opt for an iterative data collection process (e.g., theoretical or targeted sampling.^14^

The interviews were transcribed *verbatim*, pseudonymized and anonymized prior to publication. Data analysis was performed using a multi‐step coding method, informed by “Grounded Theory” methodology.^15^ Open, axial, and selective coding processes were applied subsequently.^16^ The emerging categories and subcategories concentrated on the patient's autonomy and self‐determination, related limitations and burdens, and subsequently, the patient‐CG relationship.

This study was approved by the ethics committee of the University of Luebeck (file number: 17‐288) and informed consent was sought from all participants.

## 3 RESULTS

Of 37 patients interviewed, 32 (86%) stated to have been supported by a CG during the course of the illness. We analyzed information on 41 patient‐CG relationships (32 patients with CGs and 9 CGs interviewed separately). We distinguished patient‐CG interaction patterns into “passive” (30 cases, 73%) and “active” (11 cases, 27%) regarding the respective CG's involvement in the therapy decision‐making process (see below). Patients with actively involved CGs were aged 73 to 89 (median: 80) compared to a range of 55 to 89 (median: 73) for patients with passive CG involvement. Passive CGs were present at relevant doctor‐patient conversations in 31% of cases. In cases of active CG‐involvement, the participation rate was 45%.

### 3.1 Passive caregivers

Passive CGs were supportive by addressing the needs of the patients reactively. They helped with overcoming logistical challenges, coordination of appointments, or alleviated stressful situations through their presence:

I actually just sat there and listened and asked a few questions. My wife does all that and then I got everything I wanted to know myself, right? (Husband, AM124)

These CGs were also concerned with the patient's general mental well‐being by creating a sense of normality:

…what was a priority for us was to provide encouragement. Not to make him understand that life was over now and: ‘We don't care about you any‐more’, but always to be there for him’. […] we deliberately did not want to talk about these decision‐making processes or exclusively about this topic. So, we tried, simply to keep everyday life going, to put it simply: ‘When we come (for a visit), we don't just talk about this disease’. Because we saw that that was important …(Neighbor and friend, AM107)

It should be noted that “passivity” only refers to the CG's involvement in the patient's decision‐making process and is not a value judgement and should not be understood as indifference toward the patient's illness.

### 3.2 Active caregivers

Active CGs were concerned with shaping and steering the therapy decision‐making process. They considered their strong involvement in this process as vital for adequately meeting the patient's needs. Taking into account the multitude of motives for such involvement, we expanded on the broad category of “active” CGs into two different categories: (a) patient and CG as a unit with the CGs identifying themselves with the patients' illness, and (b) reversal of the care relationship.

#### 3.2.1 Patient and caregiver as a unit: Caregivers identifying themselves with the patient's illness

Especially in cases of married couples, we observed CGs who identified themselves strongly with the patients' illness and their situation. In these couples' accounts, the aspect of experiencing the therapy process together was emphasized and perspectives were mutually shared. Accordingly, the cancer was perceived as a challenge that could only be overcome through joint efforts, and therapy decisions were always portrayed as a consensus, weighing the consequences for each individual and the relationship.

And it was clear to us that we go for the (medical) discussion and then we also went together directly, we went to the discussion, (thinking) it may be that he (the doctor) now will tell us this is now a carcinoma. And that turned out to be the case. […] We sat down at home and thought about what we'd find to be the best. And since my husband and I talk openly about the things that concern us, each of us was able to say clearly: what do I want, what (burden) am I willing carry as a wife? And basically, I said I would carry whatever (burden resulting from) how he decides. (Wife, A0119)

The extent to which a CG influences the therapy decision in these cases is difficult to determine since the eventual therapy decision is the result of a complex interpersonal and individual process. In the case cited above, the CG stated that it was the patient's decision. At the same time, the CG mentions that the burden that would have to be carried by her was also a relevant factor in the decision. The relevant steps and decisions were framed as being taken together. It can therefore be assumed that in such a patient‐CG relationship, the values, views and assessments of the CGs play a significant role in the choice of cancer therapy and the way its consequences are handled. CGs and patients thus influenced each other's perception of cancer, seeing it as a test that also conferred meaning on their relationship:

This can also create a closeness that you don't get when life goes so smoothly. No. So I have to be honest. We find that it has enriched our relationship because we have noticed how much we can give each other meaningfully in such a life or in such a difficult life situation. So that's what I think. (Wife, A0119)

#### 3.2.2 Reversal of the CG‐patient care relationship

A reversal of the care relationship between patients and their CGs could be observed when there was an apparent discrepancy in their respective skills in understanding the medical content and processes and their implications. In our sample, these CGs were always considerably younger than the patients (a difference of one generation, children or sons‐ or daughters‐in‐law). Either the patients were already having difficulties in their day to day lives owing to advanced age even before cancer diagnosis, or they had been rendered unable to act entirely independently by the acute experience of their cancer. CGs had a special responsibility here. The support to be provided by them was more comprehensive and went beyond mere support and assistance. CGs considered this form of involvement as a necessary condition for the patient to receive adequate treatment.

…the question was, the woman is over 90 or is 90, what is one to do? I tried to pave the way. Sensible diagnostics, sensible decision… (Son, AM241)

These CGs sometimes saw the need for further examination and clarification and were often initiators of the decision‐making process toward accepting treatment.

The interviews analyzed made it clear that, in particular, the coordination of medical appointments and discussions could represent a great challenge for older patients. In several cases examined, this was a central topic which also determined the role of the CGs: these patients wished to have their CG present at medical consultations, and the CGs, for their part, were convinced that their presence at medical discussions was necessary to ensure, among other things, that no information was lost, neither the relevant information about the patient to be communicated to the doctor nor that given by the doctor to the patient.

And then to the gynecologist and he discussed the therapy options with us and there I was able to discuss the questions we had considered (earlier) and so on very well. And I think that maybe that helped her once again, because I think one is simply overwhelmed in the situation, or one has so many thoughts in one's head that sometimes one can't ask questions clearly, although one would simply like to know so many things, isn't it? (Daughter, AM105)

There I had the feeling that sometimes when she went there, (she felt) also the doctor's conversations ….went too fast, right? Well, it was sometimes good when I was there, so that we could talk about it afterwards. Because sometimes it was so that I had the feeling that she was overwhelmed… when things went so fast. So (when the doctor goes on): This, this, this! […] So for that, a 10‐minute conversation or something is just a bit fast. (Daughter, AM127)

These CGs' forms of support ranged from storing, translating, and collecting medical and organizational information to extensive control over the patient's actions and decisions. Their role was offering guidance in making therapy decisions and not merely supportive, in the sense of being present and being a listener.

What is it like for an elderly person? So, here was my mother, I think – she said three Hallelujahs because I was there. So not just for psychological support, but really, yes? […] “We have to go that way now”. “And then we go now”. “Sit down there and I'll knock” and, yeah. I think that's difficult for older people in hospitals. […]

I don't think she can appreciate that. […] she doesn't even have to (Daughter, AK121)

The transformation of the CGs' role was a key observation in several interviews. This transformation has led to a shift of the CG's role in the patient‐physician‐CG triad and can be described as a role reversal: The interviews analyzed here were mostly mother‐daughter relationships. The assumption of responsibility by the CGs thus meant a reversal of the care relationship. While the “mother” bears responsibility and provides care, the “child” is freed from responsibility for itself and its actions. These CGs reported that they became aware of this role reversal when facing the necessity to make vital and immediate cancer therapy decisions. Even though they had already assumed greater responsibility for their parents or parents‐in‐law, the illness accelerated this process.

…but now that it has ‐ but it has totally changed. So, she has now put herself completely in my hands, that is the way I have to put it now. Leaves all decisions to me entirely, has also given up all power of disposal over finances, over discussions to be conducted. Takes me with her, or asks me to accompany her everywhere when she goes to doctors, which she has never done before…

(Daughter, AM130)

In one of the analyzed cases, the patient initially decided against the further treatment of her cancer. By questioning the validity and counteracting the patient's decision, the CGs assumed an active role that was decisive for the final therapeutic decision in favor of treatment. CGs thus can play a decisive role in therapeutic decisions.

Because we said: “You can't just give up now. That is not like you.” Because she hasn't done that all her life. And then I said: “Mummy, this isn't like you! You haven't explored all the options yet.” […] So I said: “it is easy to say ‘not any more’, that …” I say, “No. No. Not that.” (Daughter, AM130)

The patient had decided not to undergo further treatment. The daughter felt that this decision did not fit her life‐history and identity. The CG saw her function as contextualizing and counteracting a decision that she perceived to be irrational. In her view, she protected her mother from the consequences of a decision made under distress. She infringed the patient's autonomy to find a treatment decision consistent with her mother's values (“not giving up”). This illustrates that the concept of “autonomy” in elderly cancer patients' therapy decision making is not absolute.

## 4 DISCUSSION

The relationship between patients and their CGs can be complex and multilayered, as has been shown, for instance, by studies on patients' feelings of being a burden in the context of palliative care decision‐making.^17^ The analysis of the interviews in this study shows that the forms of support offered by CGs to their ill family members can differ considerably. We found interactions that can be described as either just supportive, defined by a mutual fate, or even a reversal of the child and parent's traditional care relationship. Taking these interaction patterns into account is important for patient‐doctor communication. By helping patients understand the information given and finding therapy options that fit their needs and wants, CGs can significantly influence patients' decision‐making and, thus, on the therapy plan's realization or failure as proposed by the oncologist. It has been shown that low levels of health literacy present challenges to any decision‐making paradigm, especially in complex cancer treatment decisions in the elderly.^18^ CGs might help the doctor define the patients' wishes and needs in terms of therapy side effects and quality of life. Analysis of the interviews also revealed that the CGs are important and sometimes indispensable for the success of therapy by assisting in organization and implementation.

When interpreting the interviews with CGs, it must be considered that not all CGs were willing to be interviewed. Forty‐six percent of CGs declined to be interviewed. The reasons given were heterogeneous, most often, no reason was given, and some CGs indicated that the situation was too stressful for them. This high rejection rate may have led to a selection of engaged CGs (bias) in our study. It is also interesting to note that although the CGs regularly criticized the health care system or organization details, which they considered needed improvement, they rarely questioned the specialist's competence as far as therapy proposals were concerned. This corresponds to the experience of the authors in oncological clinical practice. The CGs rather questioned whether a therapy concept that was generally correct was also the optimal (meaningful) one in the particular case of their family member.^8^

Although most patients (86%) indicated that they were supported by a CG, CG participation in patient‐specialist consultations was surprisingly low (38%). There is little information on whether this percentage is representative, but it corresponds to the authors' experience. We suggest that CGs should be more often integrated into the decision‐making encounters between patients and their oncologists.

The patient is the sole addressee of all medical clarifications during medical consultations. This principle is not only ethically correct but also legally enshrined. It should be preserved especially in the CG‐patient relationship we considered “care relationship reversal,” where CGs take upon themselves the responsibility for their patients because they are convinced that they know what is good or bad for the patients. Active CGs, by our definition, tended to assume that they, in particular, knew the intentions and limitations of the patients; examples are given in the results section. In the absence of CGs at patient‐doctor consultations, this situation can become particularly problematic. The patients might not have had adequate time and opportunity to express their concerns or could not participate in the discussions for various reasons, including hearing loss or speech problems. In these cases, the doctor might make a therapy proposal (or de facto decision) based on inadequate information received from the patient regarding therapy wishes. It is also possible for the patients to have misunderstood what the doctor told them. This may result in the failure of patients to pass on the physician's information correctly to the CG. Professional experience suggests that frustration on the doctor's side is especially high if agreement on therapy options is reached, but the patients later change their minds after discussing them with their CGs without further consultation with the doctor. Thus, the presence of CGs in patient‐doctor conversations can help physicians make appropriate therapy decisions in the sense of decisions that include the patients' own assessment of their conditions and wishes. However, in the case of an active CG taking over responsibility and decision‐making for the patient, the presence of a CG forming a triad in medical consultations carries the risk of the patient's individual wishes being overridden by the CG's point of view. This has been found in Asian societies^19^ and could well be true for western countries. Hence, in situations of “care relation reversal,” where the CG appears to be having total control over the patient's therapy wishes, the physician must be alert to the possibility of the patient being rendered a silent and possibly non‐consenting partner to his therapy plan. In such cases, it is necessary to actively and directly include the patient in discussions and to find an opportunity to talk to the patient alone to find out what their real wishes are.

The gold standard in patient‐doctor communication is the concept of “shared decision‐making” (SDM), which has meanwhile been revised and modernized several times.^20^, ^21^, ^22^ As early as 1997, Cathy Charles published an article with the subtitle “it takes at least two to tango.” At least two suggests that more than two may be meaningful. However, it is still not usual to involve more than the doctor and the patient in making therapy decisions. In cancer therapy, it would be particularly important not only to achieve just adherence. The patient follows the doctor's instructions, but concordance is based on the patient's conviction that the doctor's proposal is appropriate for her or him personally.^23^, ^24^ In this context, CGs can play a constructive and meaningful role by mediating between a patient and oncologist, conveying information in both directions.

## ACKNOWLEDGEMENT

Open Access funding enabled and organized by Projekt DEAL

## CONFLICT OF INTEREST

The authors declare no conflicts of interest.

## AUTHOR CONTRIBUTIONS

Conceptualization: Frank Gieseler, Andreas Heidenreich, Jacqueline Schubert, Christoph Rehmann‐Sutter, Christina Schüess, Joachim Hübner, Alexander Katalinic

Data curation: Frank Gieseler, Andreas Heidenreich, Jacqueline Schubert, Christoph Rehmann‐Sutter, Frank Wörler, Christina Schüess, Joachim Hübner, Susanne Elsner

Formal analysis: Frank Gieseler, Andreas Heidenreich, Jacqueline Schubert, Christoph Rehmann‐Sutter, Frank Wörler, Christina Schüess, Joachim Hübner, Susanne Elsner, Katarina Block, Alexander Katalinic

Funding Acquisition: Frank Gieseler, Christoph Rehmann‐Sutter, Joachim Hübner, Achim Rody, Nicolas von Bubnoff, Tobias Keck, Monica Steimann, Gero Endsin, Alexander Katalinic

Investigation: Frank Gieseler, Andreas Heidenreich, Jacqueline Schubert, Susanne Elsner, Achim Rody

Methodology: Frank Gieseler, Andreas Heidenreich, Jacqueline Schubert, Christoph Rehmann‐Sutter, Frank Wörler, Christina Schüess, Joachim Hübner, Susanne Elsner, Katarina Block, Monica Steimann, Alexander Katalinic

Project Administration: Frank Gieseler, Christoph Rehmann‐Sutter, Christina Schüess, Joachim Hübner, Achim Rody, Tobias Keck, Monica Steimann, Gero Endsin, Alexander Katalinic

Resources: Andreas Heidenreich, Achim Rody, Nicolas von Bubnoff, Tobias Keck, Monica Steimann, Gero Endsin

Supervision: Frank Gieseler, Nicolas von Bubnoff, Alexander Katalinic

Validation: Frank Gieseler, Andreas Heidenreich, Jacqueline Schubert, Fabian Frielitz, Frank Wörler, Christina Schüess, Susanne Elsner, Katarina Block, Alexander Katalinic

Visualization: Frank Gieseler, Andreas Heidenreich, Jacqueline Schubert, Fabian Frielitz, Frank Wörler, Christina Schüess, Susanne Elsner

Writing ‐ original draft: Frank Gieseler, Andreas Heidenreich, Jacqueline Schubert, Fabian Frielitz, Christoph Rehmann‐Sutter, Christina Schüess

Writing ‐ review & editing: Frank Gieseler, Andreas Heidenreich, Jacqueline Schubert, Fabian Frielitz, Christoph Rehmann‐Sutter, Frank Wörler, Christina Schüess, Joachim Hübner, Susanne Elsner, Katarina Block, Achim Rody, Nicolas von Bubnoff, Tobias Keck. Monica Steimann, Gero Endsin, Alexander Katalinic

## DATA APPROVEMENT

Andreas Heidenreiche, Susanne Elsner, Jaqueline Schubert, Joachim Huebner, Alexander Katalinic.

## TRANSPARENCY STATEMENT

Frank Gieseler is the guarantor. He declares that this manuscript is an honest, accurate, and transparent account of the study being reported; that no important aspects of the study have been omitted; and that any discrepancies from the study as planned have been explained.

## Data Availability

The data that support the findings of this study are available from the corresponding author upon reasonable request.
